# Eccentricity Constrains Spatial Working Memory Fidelity: Evidence for the Cortical Maps Hypothesis

**DOI:** 10.3390/vision10030044

**Published:** 2026-07-17

**Authors:** Siobhan M. McAteer, Anthony McGregor, Daniel T. Smith

**Affiliations:** Department of Psychology, Durham University, Stockton Road, Durham DH1 3LE, UKanthony.mcgregor@durham.ac.uk (A.M.)

**Keywords:** memory, spatial, eccentricity, precision, working memory

## Abstract

Spatial working memory (SWM) has been characterised as a flexible resource that determines the precision with which memoranda are stored. The ‘cortical map’ proposal predicts that resource allocation, and therefore mnemonic precision, is limited by the availability of cortical space to represent memoranda. This hypothesis was tested using a continuous spatial localisation task in which memory items were presented at three eccentricities and set sizes of 1–8. Localisation error increased systematically with eccentricity, as predicted by the cortical maps hypothesis. Mixture-modelling analyses indicated that the eccentricity effect was primarily driven by increases in imprecision at small set sizes (set sizes 1–5). In contrast, when set size exceeded five items, guessing made an increasingly important contribution to localisation error, suggesting that representations of peripheral locations become more vulnerable to memory failure under high memory loads. Misbinding errors were most prominent for locations nearest fixation, likely reflecting reduced inter-item spacing at small eccentricities. Stimuli were not scaled to compensate for cortical magnification, but the observed increase in imprecision closely matched predictions derived from a cortical magnification function. Together, these findings support the cortical maps hypothesis and indicate that spatial working memory representations compete for limited representational resources within the spatial maps that guide action.

## 1. Introduction

Visuospatial working memory (VSWM) is a limited capacity system for the temporary maintenance of spatial and non-spatial (visual) information about items for upcoming task completion [1]. Although there is debate surrounding how this information might be represented within VSWM, there is growing behavioural and neurophysiological support for the idea that VSWM capacity can be characterised as a finite resource that can be flexibly allocated across to-be-remembered items [2,3]. In this view, memory capacity is not constrained by a specific number of ‘slots’, but rather by the availability of memory resources to maintain representations at a functionally useful level of fidelity. The precise nature of this resource remains unclear, but one prominent idea is that resource allocation is constrained by the availability of neurons to represent the memoranda in modality-specific, topographically organised cortical maps [4,5].

The ‘cortical maps’ hypothesis proposes that each position within the map represents a value in a specific information space (e.g., colour or spatial location). Items within an information space are represented as peaks of activity within the map that compete with each other for representation. Items from different information spaces are represented in different maps and do not compete. Consistent with this idea, studies have shown single and double dissociations between feature and space in VSWM in neuropsychological patients [6,7,8] and behavioural experiments [9,10,11,12,13,14]. Furthermore, cortical areas such as the posterior parietal cortex and dorsolateral prefrontal cortex are involved in the maintenance of spatial locations, whereas non-spatial features, such as shape and orientation, are represented in more ventral pathways [15,16,17,18]. Studies using computational models have also highlighted that the properties of receptive fields can impact the precision with which items are maintained and thus decoded from VSWM. For example, when participants are required to maintain both orientation and spatial information, a retinotopic-specific representation is found throughout striate and extrastriate cortex [19]. In addition, Mackey and colleagues [20] have shown that the topographic mapping in the frontoparietal cortex, which is known to be involved in the maintenance of spatial locations, is organised by both polar angle and by eccentricity. These findings indicate that precision in VSWM, especially the maintenance of spatial locations, may be constrained by the physical properties of the cortical areas involved in the maintenance of these features.

In the case of spatial working memory, there is strong neurophysiological evidence that the cortical maps that represent spatial working memory are shared with maps used for the control of goal-directed movements such as saccades. This argument stems from the observation of retinotopic maps within motor areas, especially in those areas involved in the programming of saccadic eye movements, such as the superior colliculus, frontal eye fields (FEF) and lateral intraparietal cortex [21,22,23,24,25]. Critically, these cortical areas also maintain activation during delayed saccades [26,27], consistent with the idea that spatial working memories are maintained in cortical maps. Neuroimaging studies of human participants report shared neural representations for action, attention, and visuospatial working memory [28], and neurostimulation of regions such as FEF has consistently been shown to affect VSWM and visuospatial attention, e.g., [29,30,31,32]. The oculomotor system and VSWM appear to share a common representation, with motor maps for the generation of saccades being projected from the superior colliculus to the posterior parietal cortex for representation in a priority map that serves action, attention and VSWM [21,33,34,35,36].

Behavioural studies lend further support to the proposition that action control and VSWM share a common spatial map. Studies using the dual-task paradigm with delay-period performance of saccades have shown that memory for spatial locations is disrupted [12,37,38,39,40,41] and that this saccadic interference effect is specific to spatial memory [13]. Maintenance of a location in VSWM also results in inhibition of saccades to that location [42] and increased saccade curvature away from the remembered location [43]. Additionally, experimentally constricting the ability to plan and execute saccades significantly reduces spatial memory span [44,45]. These findings illustrate how VSWM and saccade control draw on a shared cognitive process, consistent with the idea that performing saccades creates competition for resources between saccade targets and memory items, thus reducing the availability of resources for mnemonic representations in a shared cortical map of space.

The cortical maps hypothesis is also consistent with evidence that physiological differences across the visual field are inherited by VSWM [46,47]. For example, the horizontal–vertical anisotropy in perception (reduced perceptual performance for locations along the vertical meridian compared to iso-eccentric locations along the horizontal) is present in visual working memory [48]. This anisotropy has been explained in terms of low-level physiological differences in the visual system, specifically with respect to the densities of retinal ganglion cells and cones, which are not radially symmetric across eccentricities in the visual field [49]. Similar anisotropies can be seen in shown in oculomotor behaviour, whereby large horizontal saccades have shorter latencies and higher accuracies compared to saccades along the vertical plane [50] and in spatial working memory, where spatial span and capacity (k) are significantly reduced for locations presented along the vertical meridian compared to locations presented along the horizontal meridian [51]. Furthermore, emory capacity for letters declines with eccentricity [52], and variability in memory-guided saccades increases as a function of target eccentricity [46].

To summarise, the cortical maps hypothesis argues that spatial working memory relies on topographically organised cortical maps used to represent space. The considerable overlap between neural systems involved in oculomotor control and VSWM, combined with behavioural evidence of dual-task interference between motor control and spatial working memory, is strong evidence of the view that spatial working memory relies on cortical maps that integrate spatial information in VSWM and motor representations into a single coherent representation. The cortical maps hypothesis also predicts that the fidelity of representations in VSWM will be constrained by the physiology of the cortical regions maintaining the map. However, the evidence for this position is less direct, as it draws on studies examining how perceptual anisotropies affect memory capacity, as opposed to the precision of memory representations.

One way to address this issue is to make use of the eccentricity effect in vision. It is well-documented that reaction time and detection accuracy decrease with increasing eccentricity and set size in visual search tasks [53,54] and object recognition [55]. This eccentricity effect has been explained in terms of cortical magnification, whereby there are fewer cortical resources dedicated to processing each degree of visual angle as the visual field moves further into the periphery [56]. The eccentricity effect therefore offers a more direct way to examine the cortical maps hypothesis in VSWM, in particular with respect to the key prediction that resource allocation is limited by the availability of cortical resources, which are retinotopically mapped across the visual field and decline with eccentricity. Consistent with this prediction, visual short-term memory capacity for letters declines with eccentricity [52], and variability in memory-guided saccades increases as a function of target eccentricity [46]. Notably, this effect was preserved when M-scaled stimuli were used, consistent with the idea that fewer resources were available to maintain the representation of more eccentric objects [52].

The current study examined the eccentricity effect in spatial working memory using a continuous report task to characterise the pattern of response errors in VSWM while systematically varying eccentricity and set size. If the cortical maps hypothesis is correct, an increase in localisation error with increasing eccentricity should be observed. Given RFs are less densely populated and larger at higher eccentricities, these errors should be characterised by increased imprecision.

## 2. Materials and Methods

### 2.1. Participants

A priori power analysis using G*Power v3.1.9.2 indicated that a sample of at least three participants would be needed to observe a significant main effect of eccentricity on imprecision (α = 0.05, power = 95%, effect size $\eta_{p}^{2}$ = 0.71 based on a pilot experiment—see Supplementary Materials S1). Six volunteers (Mage = 28 years, SDage = 4.24, 5 females, 1 male, 5 right-handed) were recruited from Durham University. All participants reported having normal or corrected-to-normal vision. Participants gave written consent and received compensation at a rate of £12 per session for participation. Data collection began on 20 October 2021 and ended on 7 February 2022. This experiment received ethical approval from the Durham University Psychology Department Research Ethics Committee (reference: PSYCH-2019-10-28T15:23:58-lckd86).

### 2.2. Design

We used a within-subjects design. There were two independent variables: set size (eight levels: 1–8 items) and eccentricity (three levels: 5°, 7.5°, and 10° of visual angle around central fixation). The dependent variables were localisation error, imprecision, and the probabilities of misbinding and guessing. Participants completed a total of 1440 trials across four one-hour sessions. Sessions were completed at approximately the same time on separate days. Each session comprised a practice block of 8 trials, where participants were shown the original location as well as their own response, and 360 experimental trials, with no feedback presented, randomised across 15 blocks.

### 2.3. Stimuli and Apparatus

The task was programmed using MATLAB R2019a, using the Psychophysics Toolbox [57]. The stimuli consisted of arrays comprising between one and eight coloured dots (diameter of each dot = 1° VA) and a fixation cross (height of fixation cross = 0.76° VA) positioned at the centre of the screen. The colours of each dot were chosen without repetition from a bank of eight discriminable colours: red, orange, yellow, green, cyan, blue, magenta, and purple. The visual mask comprised 800 coloured dots, like those presented at encoding, filling the annular space five to ten degrees of visual angle around central fixation. Stimuli were presented on a 20-inch CRT screen with a refresh rate of 85 Hz. Participants sat 60 cm from the computer screen, with the centre of the screen at eye level. Fixation was monitored using a tower-mounted EyeLink 1000 eye tracker (SR Research, Ottowa, ON, Canada).

### 2.4. Procedure

**Figure 1:**
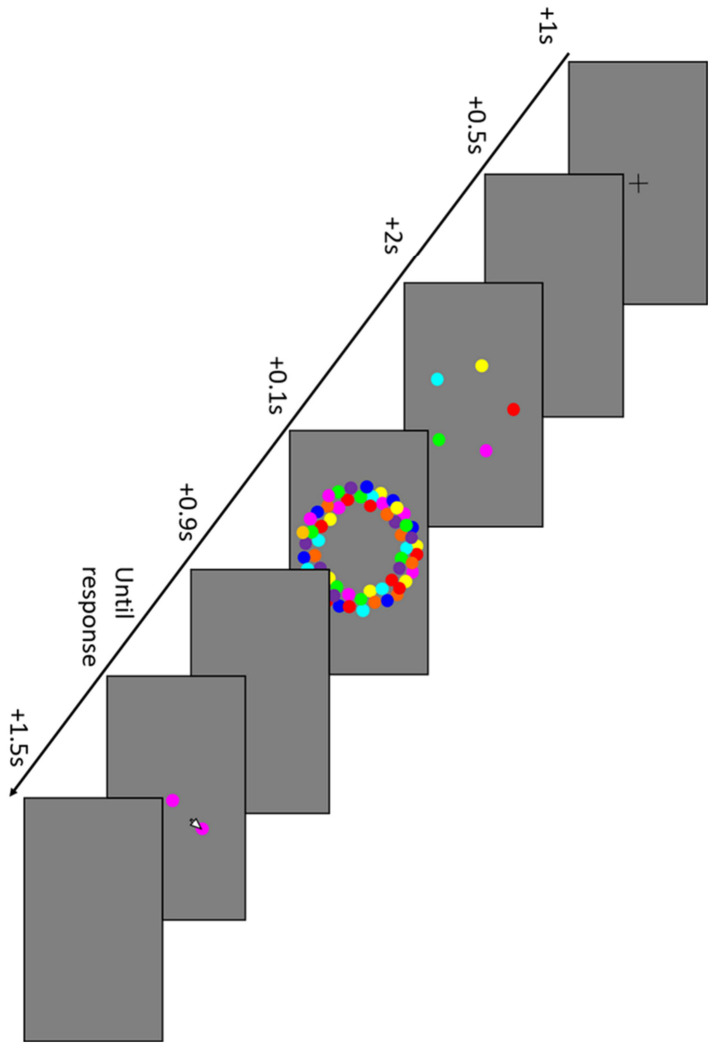
An example trial. Participants were shown an array of between one and eight coloured dots. After a short delay, they were asked to click on the screen where one of those dots first appeared.

Participants were instructed to maintain fixation throughout the trial. Trials began with presentation of a fixation cross at the centre of the screen for one second, followed by a blank screen for 0.5 s. The stimulus array, comprising between one and eight coloured dots, was then presented for two seconds. The locations of each dot were randomly chosen from eight equally spaced locations on imaginary circles with a radius of either 5°, 7.5°, or 10° of visual angle from central fixation. After presentation of the array, the visual mask was presented for 0.1 s. A blank screen was then shown for 0.9 s. At test, one of the stimuli from the array was randomly chosen and presented in the centre of the screen. Participants were required to move the mouse to click the location on screen where it first appeared. Participants could respond with any location on screen as they were not informed that the stimulus area was restricted. There was no time limit for responding. A 1.5 s blank screen followed the response period, before the beginning of the next trial. Participants were permitted to take a self-paced break between blocks. An example trial is shown in Figure 1.

## 3. Results

### 3.1. Analysis

Trials in which a saccade had an amplitude greater than 2.5° visual angle during encoding and delay were excluded from analysis (959/8640 trials; 11% of trials). When split by eccentricity, the exclusion rates were 10.6% at 5°, 11.2% at 7.5° and 11.5% at 10°. Inferential analysis was carried out in R 4.1.2 [58], using the rstatix 0.7.0 package [59]. Mean localisation error, imprecision, probability of reporting a target (PTarget) and guessing were each analysed with a 3 (eccentricity) × 8 (set size) repeated measures ANOVA. The assumption of sphericity was violated for guessing, so a Greenhouse–Geisser correction was applied to the main effect of eccentricity for this analysis. Mean misbinding was analysed with a 3 (eccentricity) × 7 (set size) repeated measures ANOVA (there are only seven levels because misbinding errors are impossible at set size 1, so this set size is omitted from the analysis).

#### 3.1.1. Localisation Error

**Figure 2:**
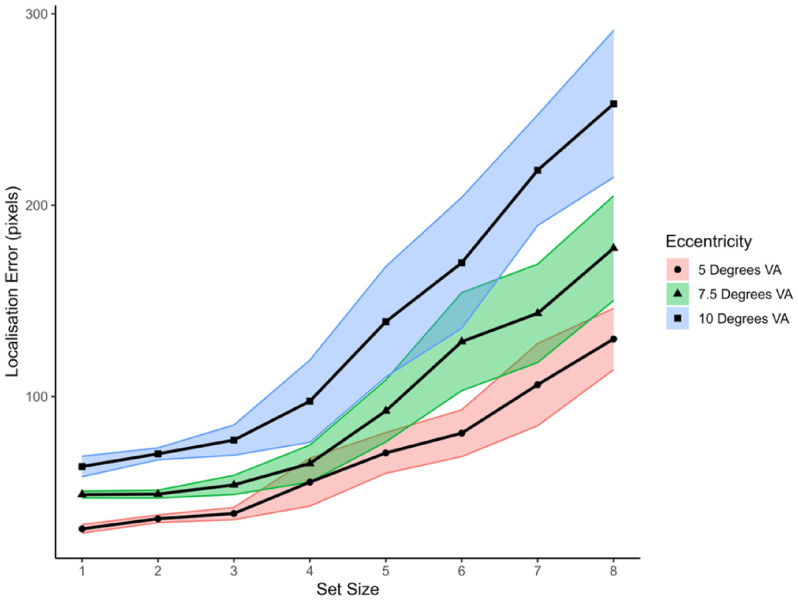
Mean localisation error at each eccentricity and set size. Filled circles (red shading) show 5°, filled triangles (green shading) show 7.5° and filled squares show 10° (blue shading). The shaded regions represent SEM.

There were significant main effects of set size (*F*_(7, 35)_ = 22.4, *p* < 0.001, $\eta_{p}^{2}$ = 0.818) and eccentricity (*F*_(2, 10)_ = 31.64, *p* < 0.001, $\eta_{p}^{2}$ = 0.864), and a significant interaction between set size and eccentricity (*F*_(14, 70)_ = 4.97, *p* < 0.001, $\eta_{p}^{2}$ = 0.498). These effects are shown in Figure 2. Examination of the differences between eccentricity conditions at each set size revealed that the difference between 5° VA and 7.5° VA was significant at set sizes 6 (*M*_5DVA_ = 77.67, *SD*_5DVA_ = 29.31; *M*_7.5DVA_ = 127.69, *SD*_7.5DVA_ = 62.4) and 8 (*M*_5DVA_ = 134.53, *SD*_5DVA_ = 38.92; *M*_7.5DVA_ = 175.58, *SD*_7.5DVA_ = 69.33); *p* ≤ 0.02. The difference between 7.5° VA and 10° VA was significant at set sizes 5 (*M*_7.5DVA_ = 91.08, *SD*_7.5DVA_ = 40.49; *M*_10DVA_ = 139.57, *SD*_10DVA_ = 68.94), 6 (*M*_10DVA_ = 166.43, *SD*_10DVA_ = 81.94), 7 (*M*_7.5DVA_ = 139.47, *SD*_7.5DVA_ = 63.97; *M*_10DVA_ = 213.3, *SD*_10DVA_ = 71.05), and 8 (*M*_10DVA_ = 251.59, *SD*_10DVA_ = 92.62); *p* < 0.001. No other differences were significant; *p* ≥ 0.07.

#### 3.1.2. Mixture Modelling

Mixture modelling [4] was carried out using MemToolbox2D [60]. The model returns four parameters: pTarget, which is the proportion of trials in which the responses are based on the target item; imprecision, which is variability in the response, operationalised as the standard deviation of the von Mises distribution centred on the veridical value (σ); the probability of guessing, which is drawn from a uniform distribution (pGuessing); and the probability of responding with a non-target (pMisbinding), which is drawn from a Gaussian centred on one of the non-probed items. Maximum likelihood estimates were obtained for these sources of recall error in each condition. The estimate of guessing was corrected by assuming that responses were sampled from the annulus within which items could appear.

#### 3.1.3. Imprecision

**Figure 3:**
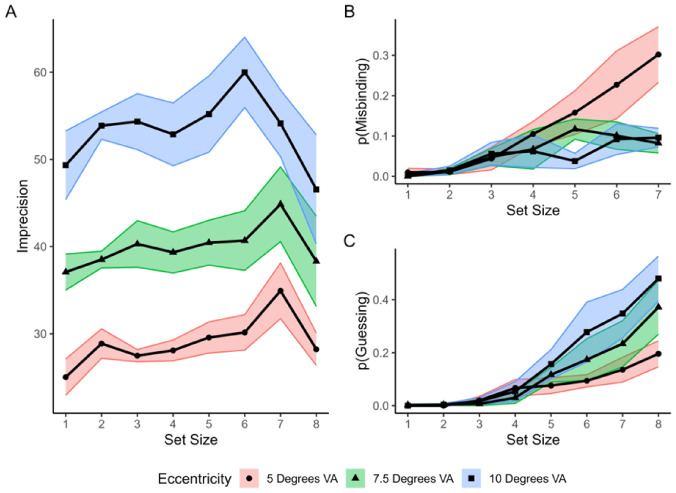
Mean imprecision (**A**), mean probability of reporting a non-target (misbinding) (**B**), and mean probability of guessing (**C**) at each eccentricity and set size. The shaded regions represent SEM.

There was a main effect of eccentricity (*F*_(2, 10)_ = 52.05, *p* < 0.001, $\eta_{p}^{2}$ = 0.689) but no main effect of set size (*F*_(7, 35)_ = 1.66, *p* = 0.155, $\eta_{p}^{2}$ = 0.004), and no interaction between eccentricity and set size (*F*_(14, 70)_ = 1.63, *p* = 0.101, $\eta_{p}^{2}$ = 0.019). These effects are illustrated in Figure 3A. Post hoc comparisons between adjacent eccentricity conditions revealed that the differences between 5° VA (*M* = 29.04, *SD* = 5.13) and 7.5° VA (*M* = 39.94, *SD* = 7.53) and between 7.5° VA and 10° VA (*M* = 53.29, *SD* = 9.87) were significant; *p* < 0.001.

To evaluate whether the observed eccentricity effect was consistent with the cortical maps hypothesis, predicted changes in imprecision were derived from the cortical magnification function proposed by Horton and Hoyt [61], M(E) = 17.3/(E + 0.75), where M represents millimetres of cortex per degree of visual angle and E represents eccentricity in degrees.

The observed increase in σ closely tracked the predicted decline in cortical representation across eccentricities. Relative to the 5° condition, imprecision (σ) increased by 40.1% at 7.5° and 87.4% at 10°, whereas the cortical magnification model predicted increases of 43% and 86%, respectively. Thus, the magnitude of the observed eccentricity effect very closely matched that expected from eccentricity-dependent changes in cortical magnification.

#### 3.1.4. pTarget

There was a significant main effect of set size (*F*_(7, 35)_ = 18.33, *p* < 0.001, $\eta_{p}^{2}$ = 0.786). Neither the main effect of eccentricity (*F*_(2, 10)_ = 2.85, *p* = 0.105, $\eta_{p}^{2}$ = 0.363) nor the interaction between eccentricity and set size was significant (*F*_(14, 70)_ = 0.75, *p* = 0.717, $\eta_{p}^{2}$ = 0.131). Bonferroni-corrected pairwise comparisons revealed that participants were significantly more likely to report the target at set size 3 (*M* = 0.97, *SD* = 0.04) compared to set size 4 (*M* = 0.91, *SD* = 0.12), at set size 4 compared to set size 5 (*M* = 0.83, *SD* = 0.14), at set size 5 compared to set size 6 (*M* = 0.74, *SD* = 0.19), at set size 6 compared to set size 7 (*M* = 0.66, *SD* = 0.21), and at set size 7 compared to set size 8 (*M* = 0.54, *SD* = 0.22); *p* ≤ 0.021. No other comparisons were statistically significant.

#### 3.1.5. Misbinding

There were significant main effects of set size (*F*_(6, 30)_ = 13.89, *p* < 0.001, $\eta_{p}^{2}$ = 0.74) and eccentricity (*F*_(2, 10)_ = 5.08, *p* = 0.030, $\eta_{p}^{2}$ = 0.504) and a set size * eccentricity interaction (*F*_(12, 60)_ = 1.94, *p* = 0.047, $\eta_{p}^{2}$ = 0.279), illustrated in Figure 3B. Examination of the differences between eccentricity conditions at each set size revealed that the difference between 5° VA and 7.5° VA was significant at set sizes 7 (*M*_5DVA_ = 0.23, *SD*_5DVA_ = 0.21; *M*_7.5DVA_ = 0.10, *SD*_7.5DVA_ = 0.08) and 8 (*M*_5DVA_ = 0.30, *SD*_5DVA_ = 0.17; *M*_7.5DVA_ = 0.08, *SD*_7.5DVA_ = 0.06); *p* ≤ 0.006. No other differences were statistically significant; *p* ≥ 0.121.

#### 3.1.6. Guessing

There was a significant main effect of set size (*F*_(7, 35)_ = 7.33, *p* < 0.001, $\eta_{p}^{2}$ = 0.284) and eccentricity Greenhouse–Geisser correction (*F*_(1.5, 7.63)_ = 5.25, *p* = 0.014, $\eta_{p}^{2}$ = 0.055). The interaction between set size and eccentricity was also significant (*F*_(14, 70)_ = 3.43, *p* < 0.001, $\eta_{p}^{2}$ = 0.078) (see Figure 3C). Analysis of simple main effects indicated guessing significantly increased with eccentricity at set size 8 (*F*_(2)_ = 7.41, *p* = 0.011) and set size 7 (*F*_(2)_ = 4.11, *p* = 0.05). The effects were non-significant at smaller set sizes (*p* > 0.08).

## 4. Discussion

The current study investigated the cortical maps hypothesis by examining the eccentricity effect in spatial working memory using a continuous report task. Consistent with this hypothesis, we observed an eccentricity effect, such that increasing eccentricity produced a systematic increase in localisation error which became greater as set size increased. Mixture modelling suggests that at set sizes 1–4, the eccentricity effect in localisation error was primarily driven by increased imprecision, but as set size increased beyond 5 items, guessing made an increasingly large contribution to localisation error. This pattern suggests that as more items compete for a limited representational resource at the same time as the available resources are reduced, the representational strength of individual items is reduced to the point where they become indistinguishable from noise and therefore irretrievable. However, given the relatively small sample, some caution should be exercised when evaluating the magnitude of the effect of the interaction between set size and guessing, and further experiments with a larger sample are needed to definitively establish the strength of this effect.

There was no evidence for a monotonic increase in imprecision as set size increased. Rather, there appears to be a small increase in imprecision as set size increased from one to two, a peak at set size 6–7, then a significant decrease in imprecision at set size 8. The majority of the monotonic increase in localisation error with set size therefore appears to be driven by increased guessing as set size increased beyond 5 items, rather than increased imprecision. This result is somewhat different to that of Schneegans and Bays [62], who reported a monotonic increase in imprecision with set size, but very similar to our previous observations [63]. The precise reason for this discrepancy is not clear. One possibility is that the study was underpowered to detect an effect of set size; however, it may also be due to the different response modes used. Specifically, we [63] used a mouse localisation task whereas [62] used a ballistic manual pointing action. The accuracy of mouse pointing responses are less affected by set size than manual pointing responses [64], which may partly explain the different response patterns. It is also noteworthy that the current experiments and [63] tested the full range of set sizes from 1 to 8, whereas [62] tested a narrower range of set sizes (1, 2, 3, 4 and 8). Given that the pattern of resource allocation in WM is known to be sensitive to task structure [65], it is possible that the differences reflect task-specific strategies adopted by participants. For example, at high set sizes, participants may have consciously decided to prioritise a smaller number of items to ensure accurate responding on this subset of items rather than attempting to store all items at a low level of precision. This strategy would produce relatively stable levels of imprecision but at the cost of increased guessing as set size increased, as we observed.

Misbinding was greatest for the stimuli closest to fixation. It seems likely that this effect is an artefact of the decreasing spacing between stimuli at large set sizes. At small eccentricities, the spacing may have been sufficiently low that some ‘guesses’ landed unintentionally close to the location of a non-target item, thus inflating misbinding. The stimuli were not scaled with eccentricity, so participants were unlikely to unintentionally place a guess on the position of a non-target item when arrays were presented at the more eccentric locations, even at larger set sizes.

There are some limitations to our study. Firstly, although adequately powered to detect main effects of eccentricity, the study may have been underpowered to detect weaker but still meaningful interactions. The small sample of young participants drawn from a university population also means some caution is required in generalising to other populations, such as older people. Furthermore, as noted earlier, the stimuli were not scaled to compensate for changes in cortical magnification across eccentricities. Consequently, it is not possible to determine whether the observed increase in imprecision originated during perceptual encoding, memory maintenance, or both. However, the magnitude of the eccentricity effect closely matched those predicted by a cortical magnification function. This correspondence suggests that the decline in spatial working memory fidelity reflects constraints imposed by the organisation of retinotopic cortical maps, although future studies employing cortical scaling will be required to establish this more definitively.

Overall, these data are consistent with the cortical maps hypothesis of SWM [5] and the more specific claim that spatial working memory is represented in the same retinotopically organised cortical representations used to guide visually directed actions such as eye movements [12,14,28,38,42,43,44,66]. Localisation error increased systematically with eccentricity, and mixture-modelling analyses indicated that this effect reflected both increases in mnemonic imprecision and increases in guessing. Notably, the magnitude of the effect of eccentricity on imprecision closely corresponded to that predicted by reductions in cortical magnification. Together, these findings are consistent with the view that the fidelity of spatial working memory representations is constrained by the properties of the underlying cortical maps. Indeed, the idea that visually guided actions and spatial working memory share a representation is a computationally efficient way to temporarily maintain information for upcoming task completion. This eccentricity effect aligns with the broader theoretical position that the principal functional role of VSWM is the control of action [67,68,69].

The observation of an eccentricity effect in spatial working memory is also broadly consistent with sensory recruitment hypotheses, which argue that cortical areas involved in perception are also involved in maintenance of VSWM representations [70,71]. This interpretation draws on evidence for shared neurophysiological mechanisms underlying perception and memory [72,73,74] and behavioural evidence that crowding occurs in both VSWM and perception [75]. Although the present findings do not directly demonstrate shared neural substrates, the dependence of memory performance on eccentricity is consistent with the idea that visual–spatial working memory inherits constraints from the organisation of perceptual cortical maps.

## 5. Conclusions

We found an eccentricity effect in spatial working memory such that localisation error increased with eccentricity. This effect became larger as set size increased. Analysis of the sources of error with mixture modelling showed that imprecision and guessing increased with increases in eccentricity. When set size exceeded five items, guessing, rather than imprecision, became the main driver of localisation error. Given that the availability of cortical resources for encoding spatial locations declines with increasing eccentricity, these data suggest that the allocation of VSWM resources is constrained by the neural architecture of the visual system. These results converge with neurophysiological evidence for shared cortical representations for action goals and memorised spatial locations, neuropsychological evidence that lesions to brain areas involved in visually guided actions also disrupt spatial working memory and behavioural evidence that dual tasks that involve visually guided actions disrupt spatial working memory. There was no monotonic increase in imprecision with set size, which may be due to participants’ adoption of conscious mnemonic strategies that prioritise the encoding of fewer items at a higher level of precision. Together, these findings support the idea that spatial working memory representations compete for limited representational resources in the spatial maps used to control action, as hypothesised by the cortical maps hypothesis.

## Data Availability

The data presented in this study are openly available in OSF at https://osf.io/y864x/ (accessed on 15 July 2026).

## Supplementary Materials

The following supporting information can be downloaded at: https://www.mdpi.com/article/10.3390/vision10030044/s1, Figure S1: Mean localisation error (in degrees VA) as a function of set size for each eccentricity. S1: Pilot Data.
