# Supplementary material for: Eccentricity Constrains Spatial Working Memory Fidelity: Evidence for the Cortical Maps Hypothesis
Source: Vision (Basel). 2026 Jul 17;10(3):44. doi: 10.3390/vision10030044 (PMC13398193; doi:10.3390/vision10030044)
Supplement: Supplementary file 1 [file vision-10-00044-s001.zip › vision-4402320-supplementary.pdf]

Supplementary Materials: Eccentricity Constrains Spatial Working Memory Fidelity: Evidence for the Cortical Maps Hypothesis

Siobhan M McAteer, Anthony McGregor and Daniel T Smith*

S1: Pilot Data

Methods

Participants

Fifteen participants (*M* = 22.67 years, *SD* = 6.66, 12 females, 3 males, 15 right-handed) volunteered. All participants reported having normal or corrected-to-normal vision. Participants received credit for taking part. This experiment received ethical approval from the Durham University Psychology Department Research Ethics Committee (reference: PSYCH-2019-10-28T15:23:58-lckd86).

Design

We used a within-subjects design. There were two independent variables: set size (eight levels: 1-8 items) and eccentricity (three levels: 5°, 7.5°, and 10° of visual angle around central fixation). The dependent variable was localisation error. Participants completed 8 practice trials before completing the experiment. There were 360 experimental trials, randomised across 15 blocks.

Stimuli and apparatus

The task was programmed using MATLAB R2019a, using the Psychophysics Toolbox [57]. The stimuli consisted of arrays comprising between one and eight coloured dots (diameter of each dot = 1° VA) and a fixation cross (height of fixation cross = 0.76° VA) positioned at the centre of the screen. The colours of each dot were chosen without repetition from a bank of eight discriminable colours: red, orange, yellow, green, cyan, blue, magenta, and purple. The visual mask comprised 800 coloured dots, like those presented at encoding, filling the annular space five to ten degrees of visual angle around central fixation. Participants' gaze was monitored using a tower-mounted EyeLink 1000 eye tracker (SR Research). Stimuli were presented on a 20-inch CRT screen with a refresh rate of 85Hz. Participants sat 60 cm from the computer screen, with the centre of the screen at eye level.

Procedure

Participants were instructed to maintain fixation on the centre of the screen throughout each trial. Trials began with presentation of a fixation cross at the centre of the screen for one second, followed by a blank screen for 0.5 s. The stimulus array, comprising between one and eight coloured dots, was then presented for two seconds. The locations of each dot were randomly chosen from eight equally spaced locations on imaginary circles with a radius of either 5°, 7.5°, or 10° of visual angle from central fixation. After presentation of the array, the visual mask was presented for 0.1s. A blank screen was then shown for 0.9s. At test, one of the stimuli from the array was randomly chosen and presented in the centre of the screen. Participants were required to move the mouse to click the location on screen where it first appeared. Participants could respond with any location on screen as they were not informed that the stimulus area was restricted. There was no time limit for responding. A 1 s blank screen followed the response period, before the beginning of the next trial. Participants were permitted to take a self-paced break between blocks.

Results

Trials in which average saccade amplitude exceeded two degrees of visual angle were excluded from analysis. This resulted in three datasets being removed from the analysis. Of the remaining 12 datasets, 39.1% of trials were removed from the analysis. The mixture model [4,60] was fit to the data to gain an estimate of effect size for the main effect of eccentricity on imprecision.

Eccentricity x set-size repeated-measures ANOVA revealed significant main effects of set size [$F(3.04, 33.42) = 3.63$, $p = .022$, $\eta_p^2 = .248$] and eccentricity [$F(1.33, 14.67) = 26.45$, $p < .001$, $\eta_p^2 = .706$]. The interaction between set size and eccentricity was not significant; $F(14, 154) = 1.08$, $p = .375$, $\eta_p^2 = .090$.

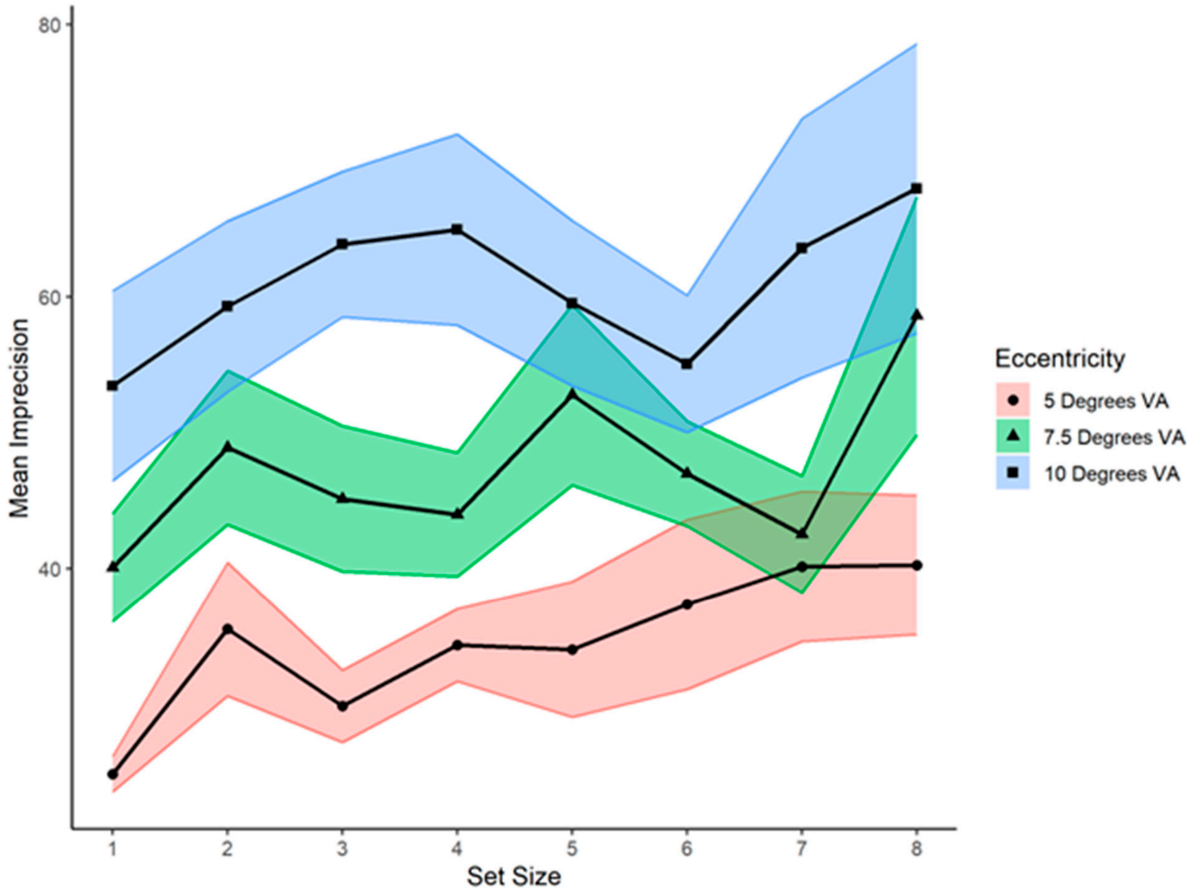

Figure S1. Mean localisation error (in degrees VA) as a function of set size for each eccentricity. The shaded regions represent SEM.

Holm–Bonferroni corrected pairwise comparisons between eccentricity conditions revealed significant differences between 5° VA (M = 34.56, SD = 15.73) and 7.5° VA (M = 47.38, SD = 19.53; $p < .001$) and between 7.5° VA and 10° VA (M = 60.96, SD = 24.96; $p < .001$). The difference between 5° VA and 10° VA was also significant; $p < .001$.

Holm–Bonferroni corrected pairwise comparisons between set size conditions revealed a significant difference between set size one (M = 39.47, SD = 19.76) and set size two (M = 47.92, SD = 21.4) only; $p = .007$. No other differences were significant; $p \geq .906$.

References

4. Bays, P.M.; Catalao, R.F.G.; Husain, M. The precision of visual working memory is set by allocation of a shared resource. *J. Vis.* **2009**, *9*, 7.

57. Kleiner, M.; Brainard, D.H.; Pelli, D.; Ingling, A.; Murray, R.; Broussard, C. What's new in Psychtoolbox-3. *Perception* **2007**, *36*, 1–16.

60. Grogan, J.P.; Fallon, S.J.; Zokaei, N.; Husain, M.; Coulthard, E.J.; Manohar, S.G. A new toolbox to distinguish the sources of spatial memory error. *J. Vis.* **2020**, *20*, 6. https://doi.org/10.1167/jov.20.13.6.
